# Supplementary material for: Concomitant opening of a bulk-gap with an emerging possible Majorana zero mode
Source: Nat Commun. 2019 Apr 29;10:1940. doi: 10.1038/s41467-019-09771-0 (PMC6488617; doi:10.1038/s41467-019-09771-0)
Supplement: Supplementary file 1 — Supplementary Information [file 41467_2019_9771_MOESM1_ESM.pdf]

Supplementary information for "Concomitant opening of a bulk-gap with an emerging putative Majorana zero mode"

Grivnin et al.

Here, we present additional data, measured in other devices and on the device used in the main text after thermal recycling. In addition, we show the tunneling conductance in other regimes of the chemical potential and the magnetic field.

Supplementary Figures

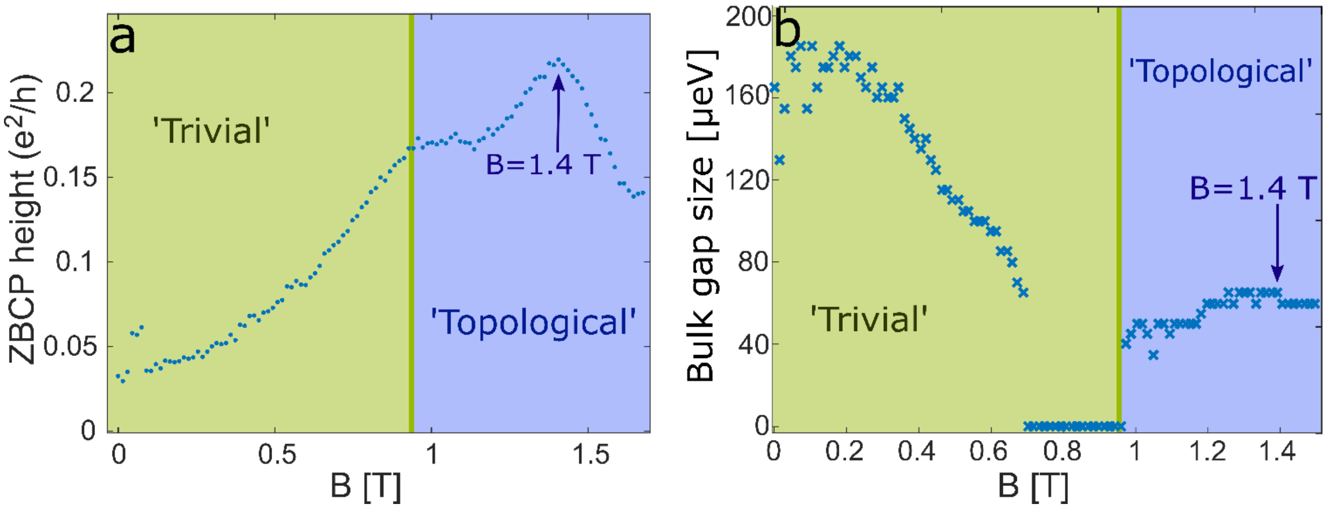

Supplementary Figure 1. ZBCP and bulk-gap as function of Zeeman field a) ZBCP – a line-cut at zero bias from Fig. 2a. **b)** bulk-gap calculated from Fig. 2b – both are function of the Zeeman field of the device presented in the main text. At the trivial region, *B*<0.95T, the gap decays with magnetic field while the ZBCP height rises. At the topological region, the ZBCP height increases with *B*, reaching a maximum where the topological gap is maximal (*B*~1.4T). For higher magnetic fields both ZBCP and the bulk-gap decay with *B* due to the softening of the superconductivity. For magnetic fields higher than 1.5T the gap shoulders smear away and the calculation of the gap size is unreliable.

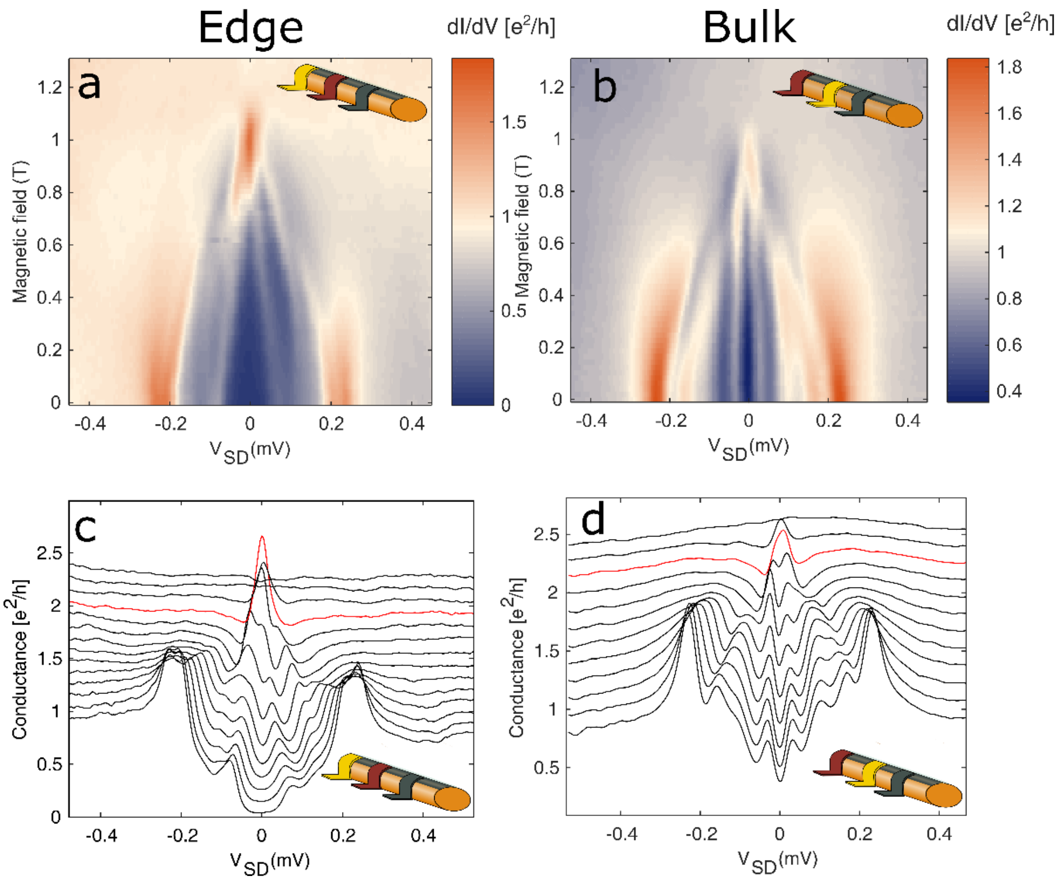

Supplementary Figure 2. Simultaneous measurement of bulk and edge of device #2.

In this device we didn't observe the reopening of the bulk gap (only its closure at *B*~0.9T, **b** & **d)**; while a strong ZBCP appear at *B*~0.9T **(a** & **c)**, to remain with a conductance ~1.8e^2/h (near the expected quantized value). Here, there was a finite misalignment of the nanowire with respect to the direction of the magnetic field, ~7 degrees. This resulted in a finite component of the magnetic field perpendicular to the epitaxial Al, which caused a reduction in the critical field from *B*=1.7T to *B*=1.2T, thus limiting the ability to see the gap reopening. The back-gate voltage is V_{BG}=-3.3V. The line traces in c & d are with magnetic field shift of 100mT.

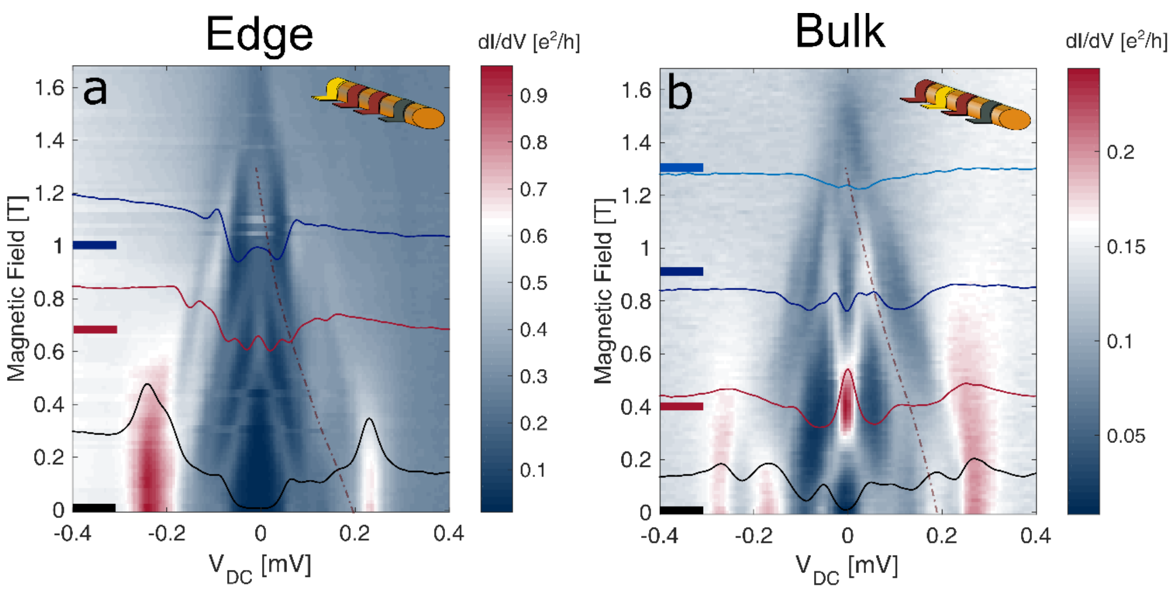

Supplementary Figure 3. Tunneling conductance in a temperature-recycled device. a) The edge-TP **b)** The bulk-TP. Cuts of the 3D plots at three different magnetic fields (marked by thick lines on left axes) are drawn on top (black, red, blue, cyan). **a)** Edge-TP shows a ZBCP, which appears near B=0.6T and survives until superconductivity diminishes at B=1.6T. **b)** The bulk-TP shows gap closing near B=0.3T and reopening at B=0.6T, with a maximal gap size of 40μeV at 0.92T. The ZBCP at the edge appears right after the gap reopens in the bulk. An Andreev state existing both at the edge and in the bulk, marked at positive bias by a red dotted line. As the latter states merge at zero-bias, the ZBCP widens - suggesting level-repulsion that is smeared by the tunnel-broadening. The back-gate voltage is V_{BG}=-4.57V.

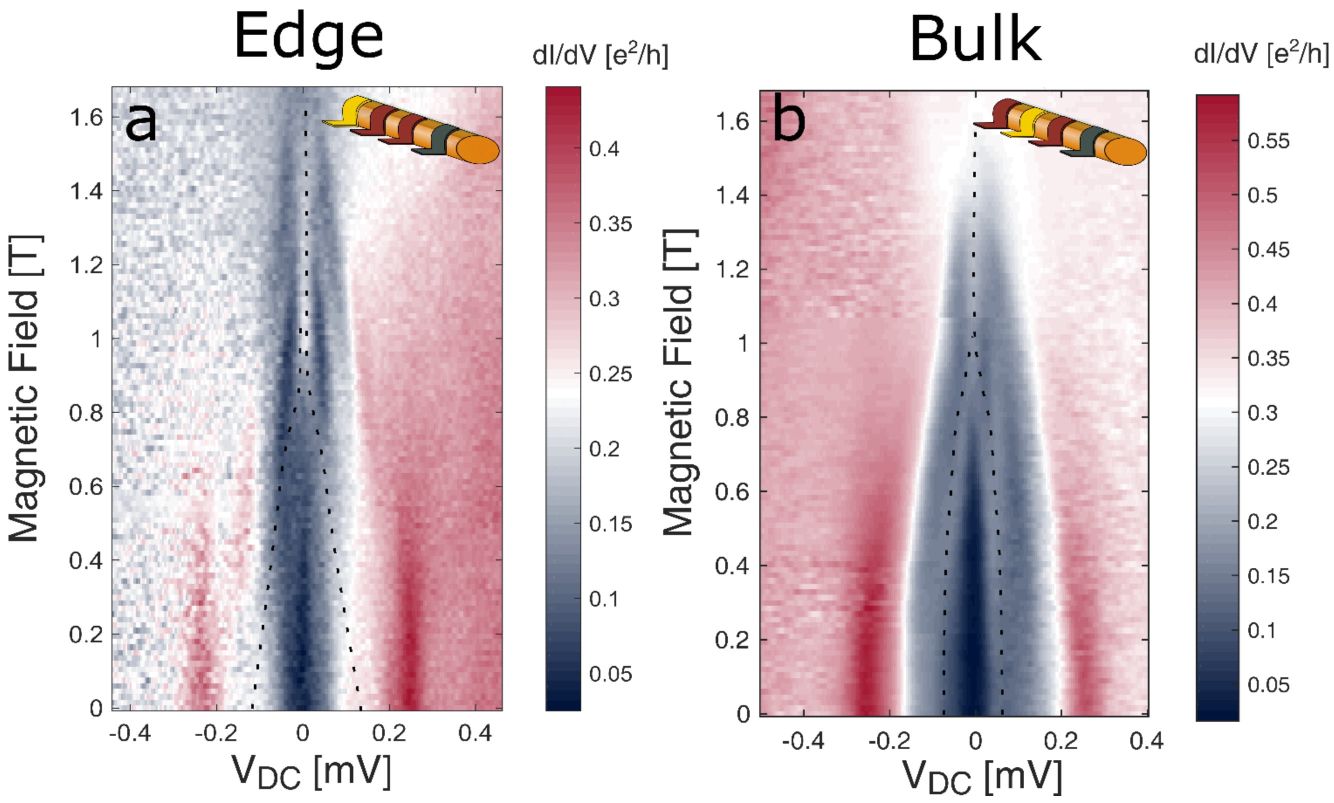

Supplementary Figure 4. ZBCP at the edge of the nanowire with zero bulk gap. This is a complimentary measurement to Fig. 3 in the main text. Here, the chemical potential is tuned away from the 'sweet spot', thereby lowering the chemical potential. The results measured on the same device but with different back-gate voltage, V_{BG}=-4.29V. **a)** The edge-TP shows a ZBCP, which appears at B~1.0T and lasts until superconductivity diminished around B~1.7T. **b)** The bulk-gap closes around B=1.0T and doesn't reopen with magnetic field (or maybe very small and below our resolution); a signature of a non-topological phase in the nanowire.

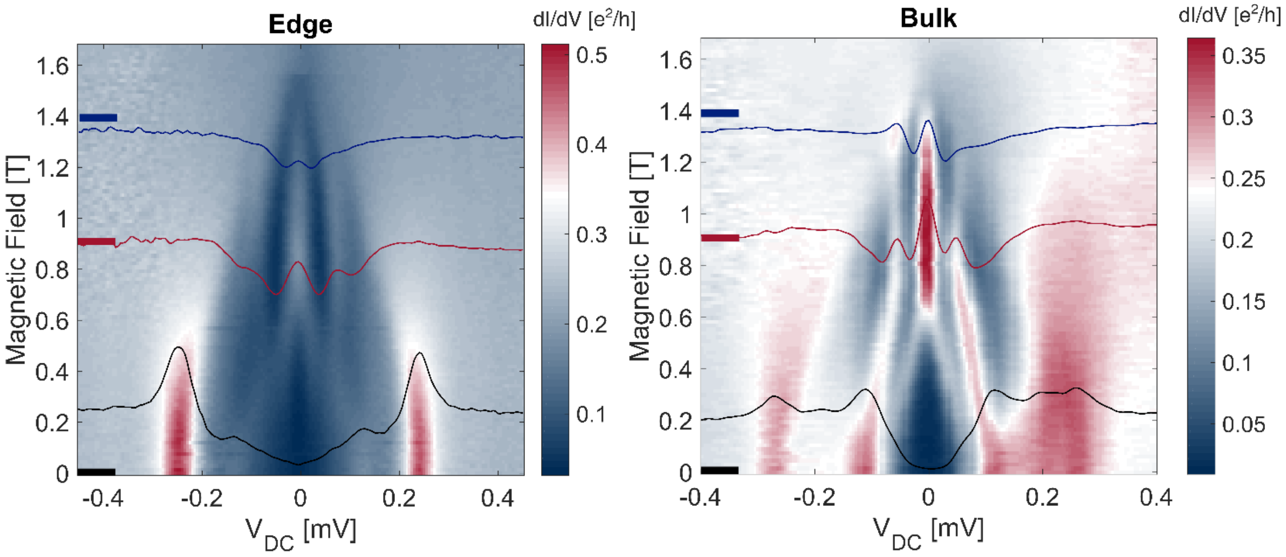

Supplementary Figure 5. Trivial ZBCP at the edge of the nanowire, in a temperature recycled device. a) Edge-TP shows a ZBCP appearing at B~0.7T and lasting until superconductivity diminished. **b)** The bulk-gap closes around B=0.7T and does not reopen with magnetic field (or maybe very small and below our resolution); a signature of a non-topological phase in the nanowire. Here, V_{BG}=-4.59V.

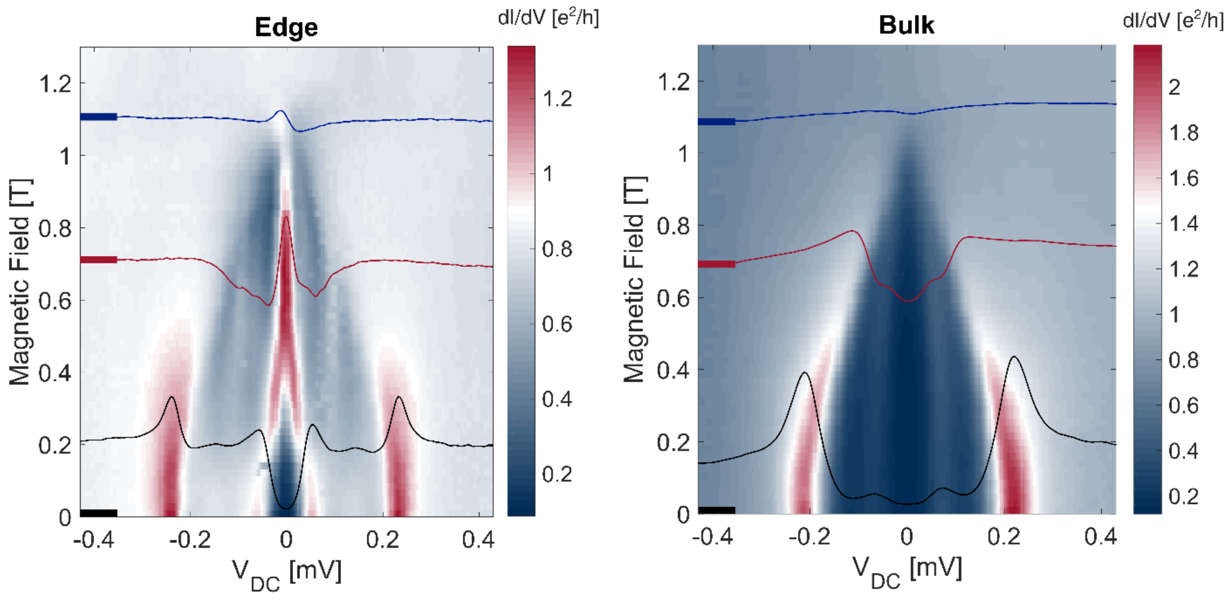

Supplementary Figure 6. Trivial ZBCP at the edge of the nanowire, in device #2 (small angled device). a) Edge-TP shows a ZBCP, which appears at *B*~0.5T and lasts until superconductivity diminished. **b)** Trivial bulk with no gap closure; a signature of a non-topological phase in the nanowire. Here, V_{BG}=-3.92V.

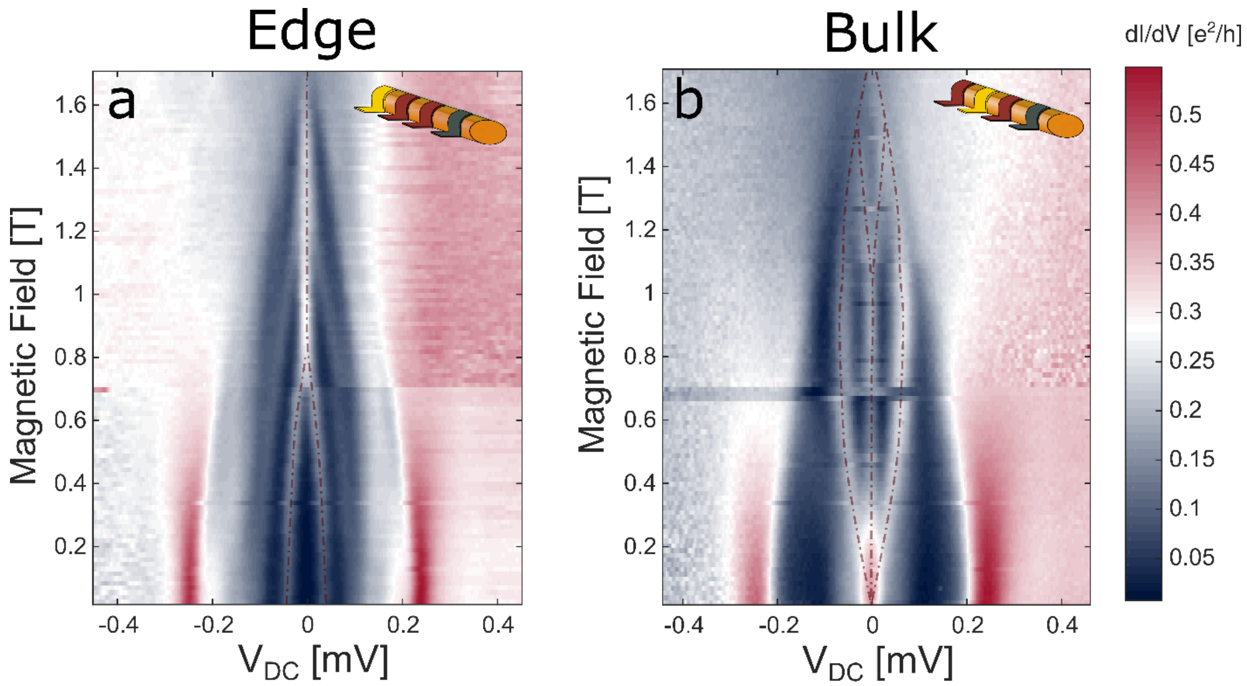

Supplementary Figure 7. Non-topological ZBCP: Edge and bulk show ZBCP.

Another example of a ZBCP in the same device used in the main text, but with a trivial bulk, at V_{BG}=-4.91V, far from the topological transition. **a)** The ZBCP appears at *B*~0.8T, persisting until superconductivity dies out; **b)** The bulk-TP shows a zero-bias state at zero magnetic field. The figure also shows a *B* dependent Andreev bound state, which splits with magnetic field in addition to another state at zero bias. The latter peak remains throughout nearly the entire range of magnetic field, splitting finally at *B*~1.1T. In this configuration, even though a ZBCP emerges at the edge, the bulk is not gapped (below *B*~1.1T), and the gap opens only at around *B*~1.1T. A possible explanation for the absence of this state at the edge is that the wire is partly depleted, and thus the potential landscape is weakly screened, with the two tunnel-probes weakly uncorrelated

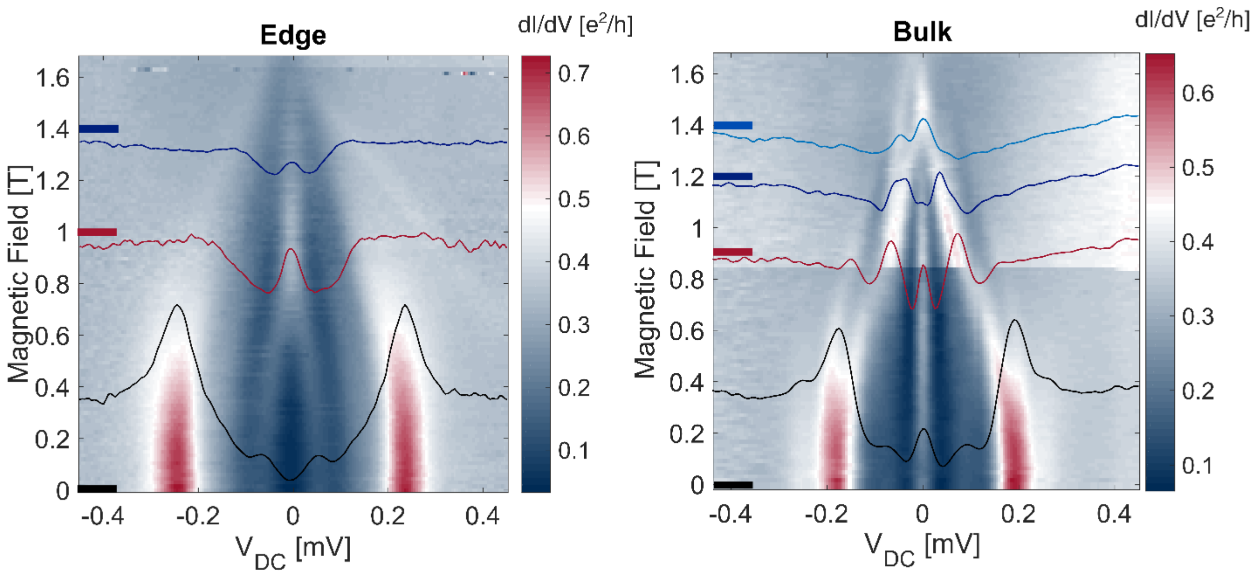

Supplementary Figure 8. Trivial ZBCP at the edge of the wire, in a temperature recycled device. a) The edge-TP. **b)** The bulk-TP. Cuts of the 3D plot at three different magnetic fields (marked by thick lines on left axes) are drawn on top (black, red, blue, cyan). **a)** The edge-TP shows a ZBCP, which appears at *B*~0.8T and survives until superconductivity diminishes at 1.6T. **b)** The bulk-TP shows a zero-bias state from zero to high magnetic field.

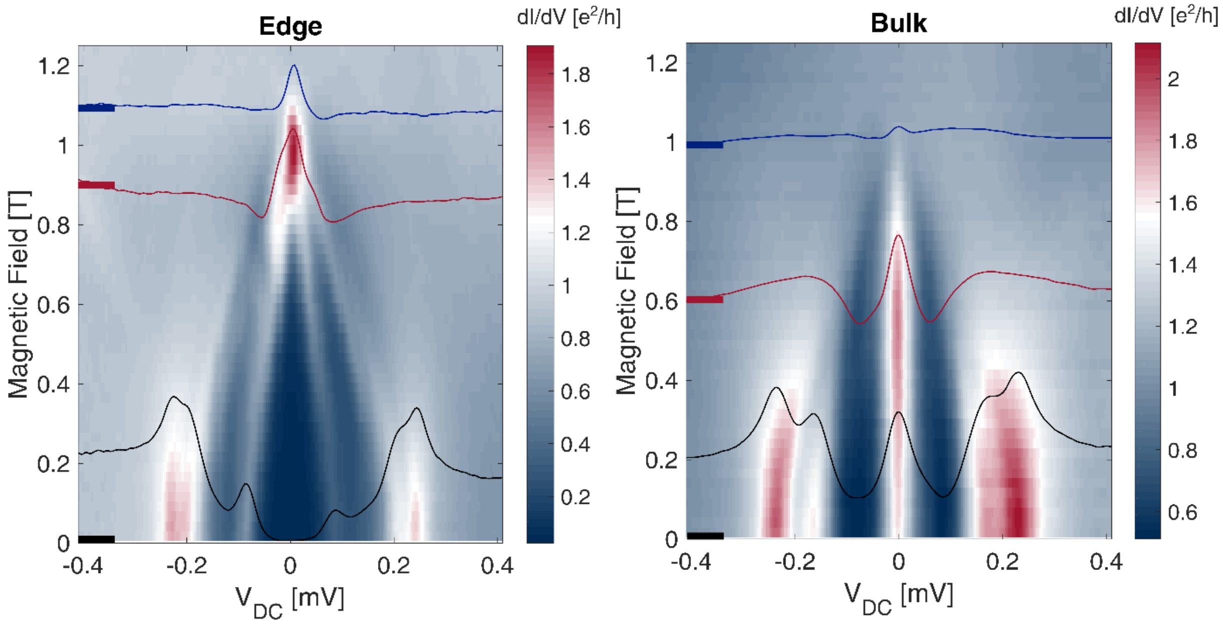

Supplementary Figure 9. Trivial ZBCP at the edge of the wire, in device #2 (small angled device). a) The edge-TP. **b)** The bulk-TP. Cuts of the 3D plot at three different magnetic fields (marked by thick lines) are drawn on top (black, red, blue). **a)** The edge-TP shows a ZBCP, which appears at *B*~0.9T and survives until superconductivity diminishes at 1.2T. **b)** The bulk-TP shows a zero-bias state from zero to high magnetic field.

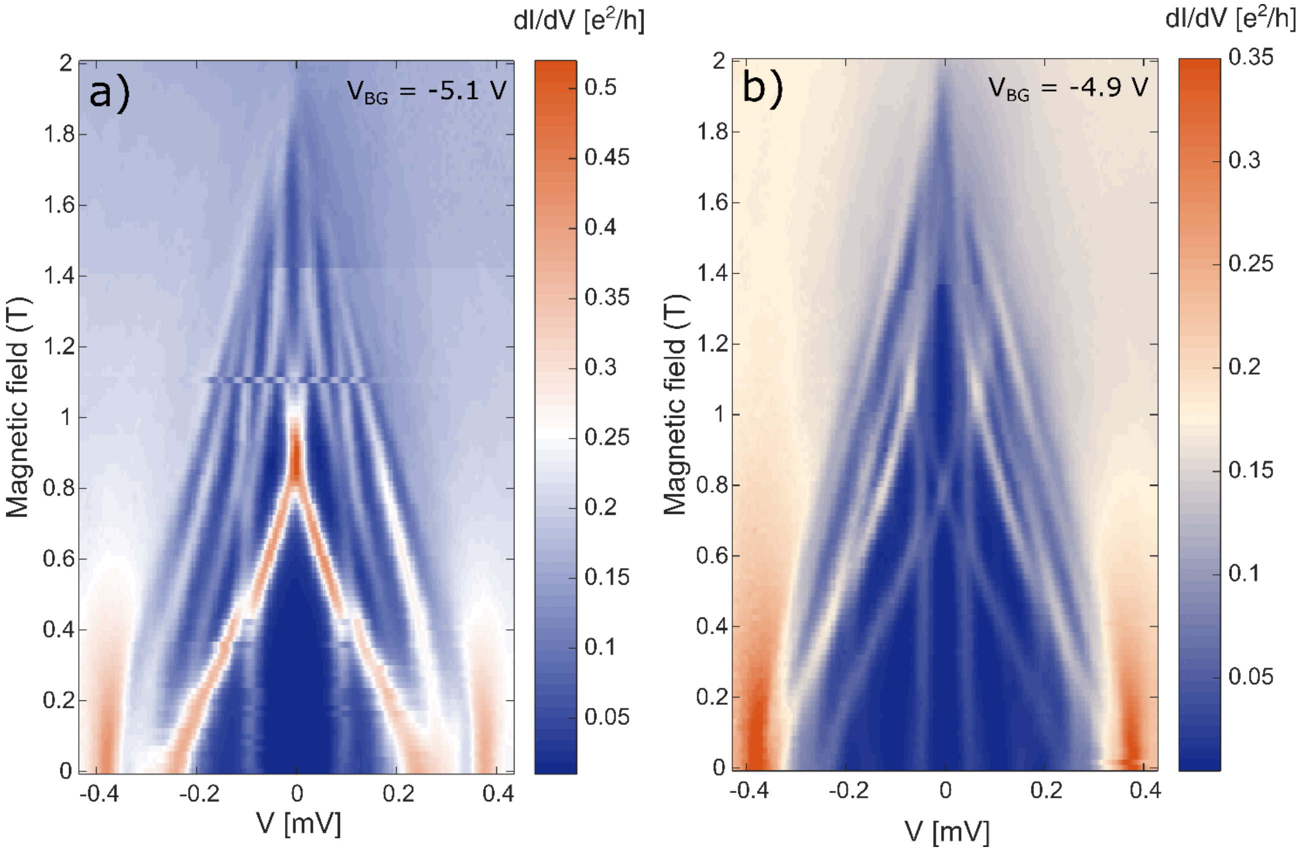

Supplementary Figure 10. Rich structure in the bulk at low density.

The tunnel probes utilized for the experiment were developed especially for the purpose of studying the bulk and the edge of the InAs nanowires, aiming to have weak tunneling coupling (but not too weak). Here, we show measurements of devices with only bulk-TP (resistance ~140kΩ). For a certain range of back-gate voltage a gap closer and reopening is seen. Several sub-gap states are visible and avoided crossing is observed between two pairs. **a)** V_{BG}=-5.1V and **b)** V_{BG}=-4.9V.

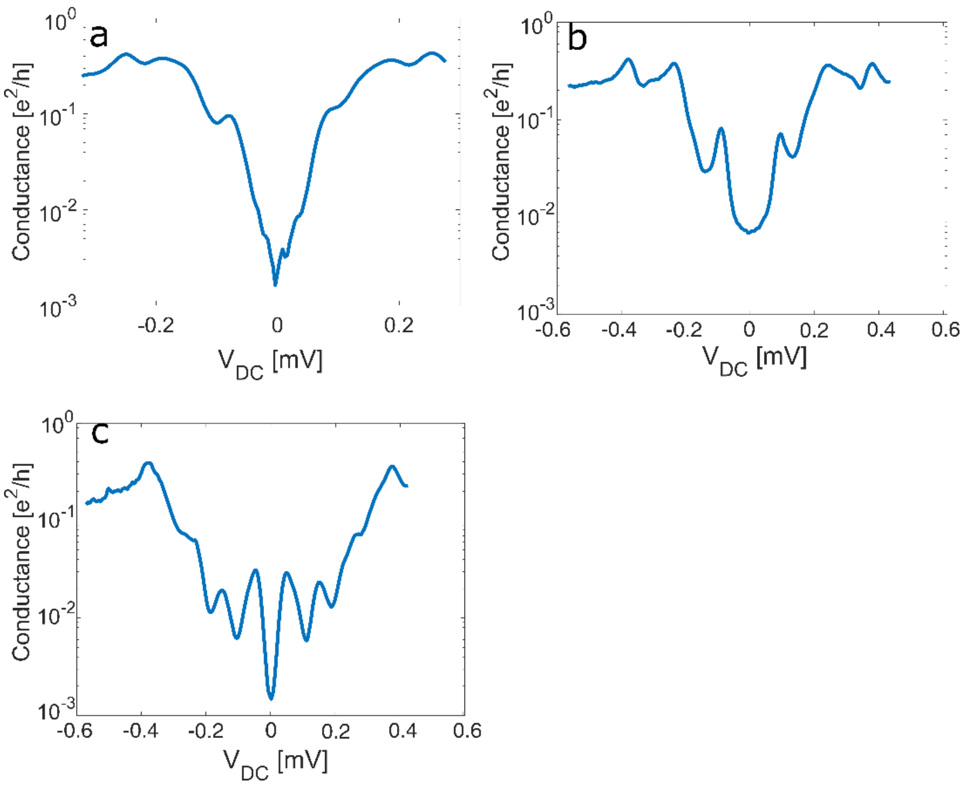

Supplementary Figure 11. Log - scale conductance.

Log - scale of the conductance measured by TP's at zero magnetic field. **a)** Main device, conductance of bulk-TP shown in Fig. 1d. **b, c)** Bulk-TP corresponding to **Supplementary Fig. 10**.

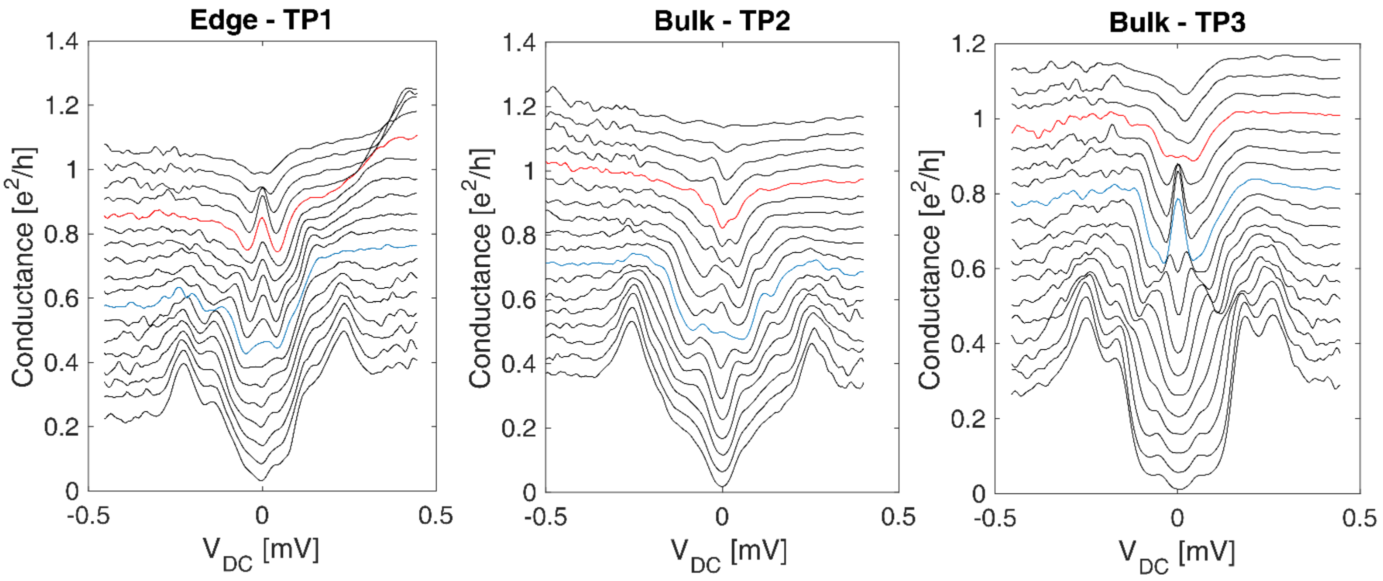

Supplementary Figure 12. Waterfall plots of device #1 (Complimentary data for Fig. 2). Conductance plots of the edge **a)** and two bulk-TPs **b** & **c)** presented in Fig. 2 in the main text. In panels **a)** and **b)** the blue curve marks the conductance at gap closure and red curve the gap reopening in bulk TP2. In panel **c)** the blue curve marks the conductance at gap closure and red curve the gap reopening in bulk TP3. The line traces are with magnetic field shift of 100mT.

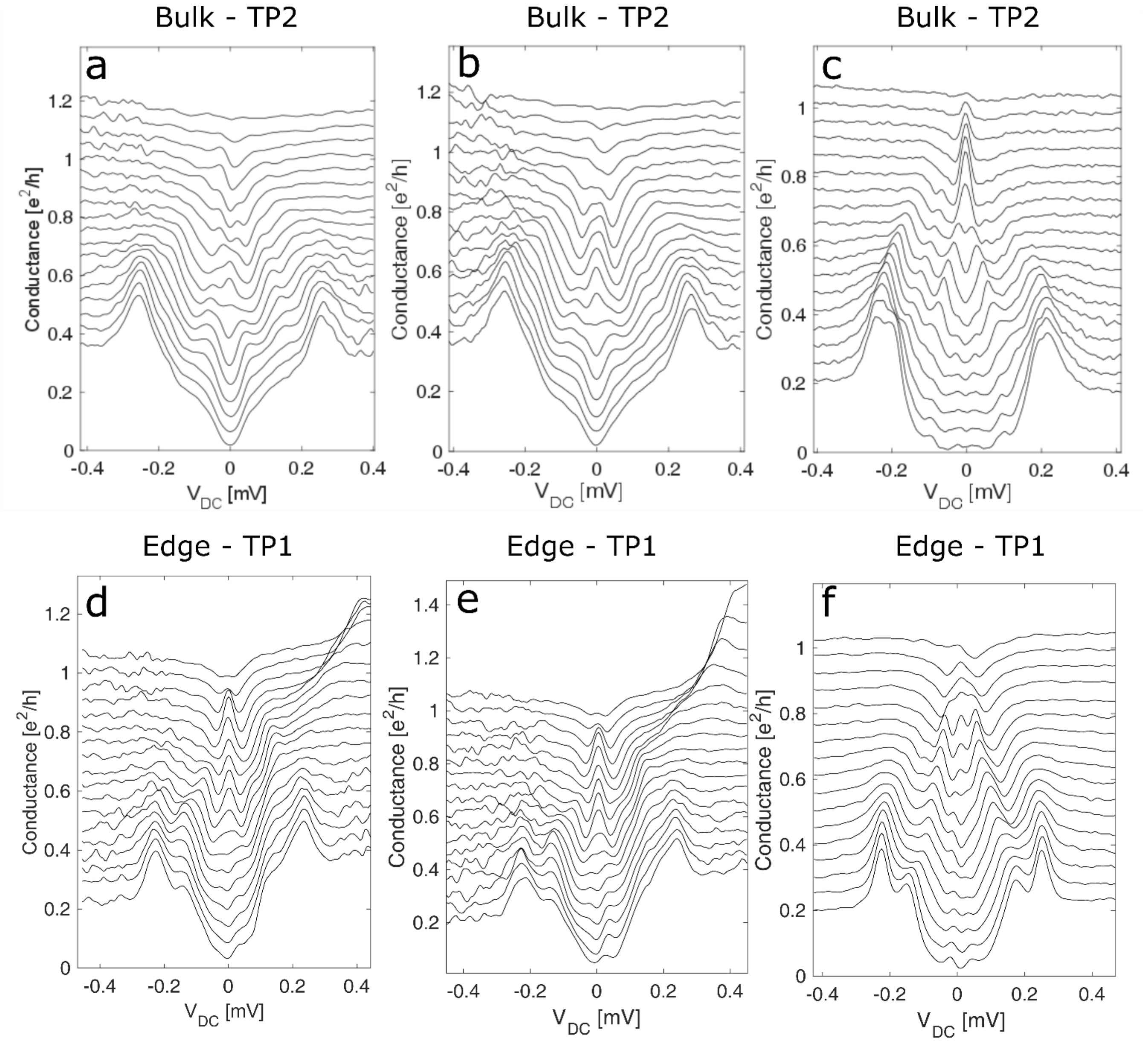

Supplementary Figure 13. Waterfall plots – correlation between bulk and edge (Complimentary data for Fig. 3).

Conductance plots of the bulk-TP **a)-c)** and Edge-TP **d)-f)** at different back-gate voltages as presented in Fig. 3 in the main text. The chemical potential is tuned by the back-gate voltage: V_{BG}=-4.22V (a, d), V_{BG}=-4.21V (b, e), V_{BG}=-4.13V (c, f). The line traces are with magnetic field shift of 100mT.

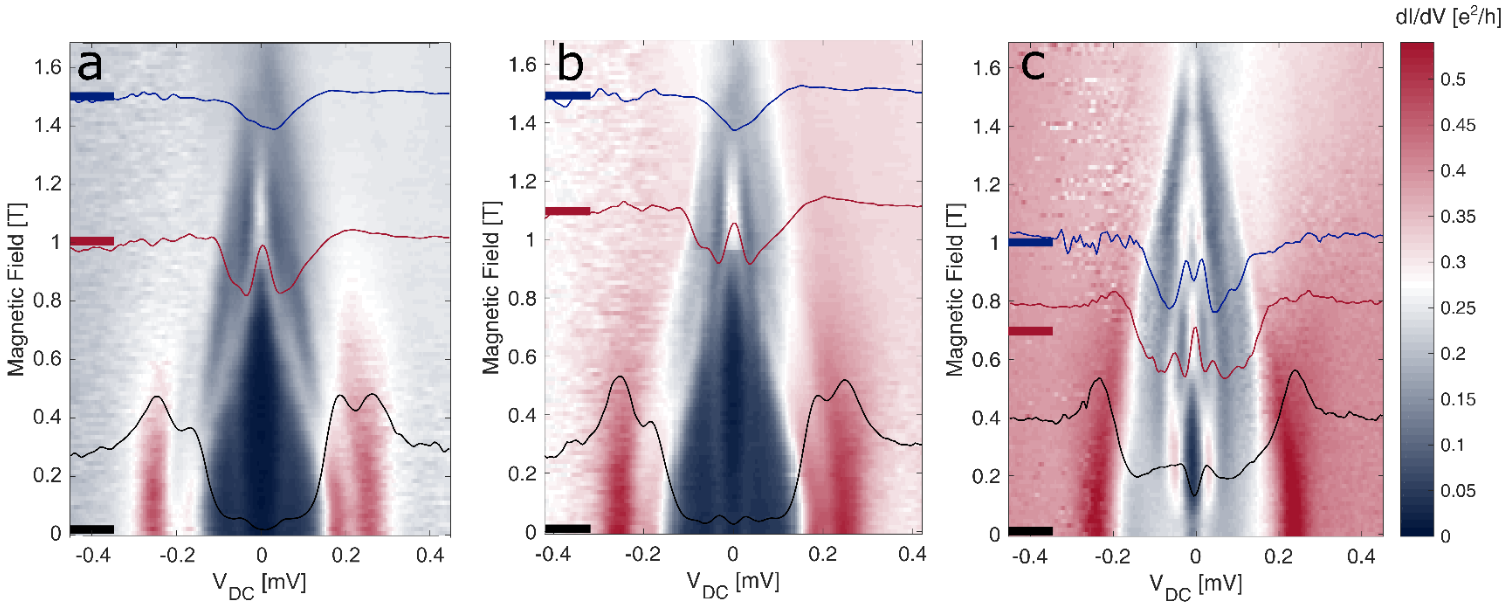

Supplementary Figure 14. TP3 dependence on V_{BG} (Complimentary data for Fig. 3).

This is another complimentary measurement to Fig. 3 in the main text. Spectroscopy of the bulk as function of back-gate voltage with TP3. The chemical potential is tuned by the back-gate voltage: V_{BG}=-4.22V **a)** V_{BG}=-4.21V. **b)** V_{BG}=-4.13V. **c)** Cuts of the 3D plot at three different magnetic fields (marked by thick lines on left axes) are drawn on top (black, red, blue). **a)** The chemical potential is in the 'sweet spot' in TP1 and TP2 and slightly shifted in TP3. Here, bulk gap reopens at B~1.4T, reaching E_g~30µeV. **b)** Gap closing without reopening. **c)** Gap closing at 0.7T, reopening at 1.0T and closing again at 1.3T.

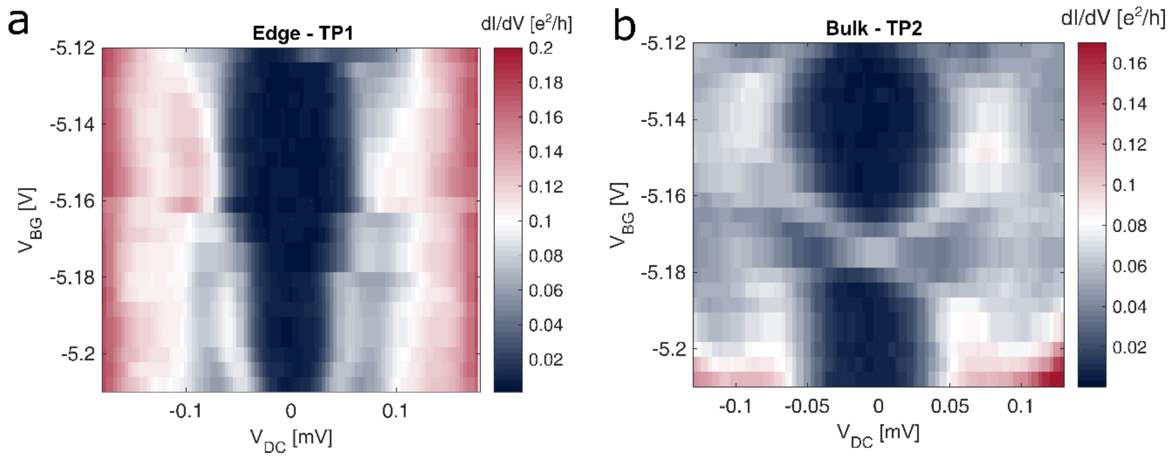

Supplementary Figure 15. Trivial behavior of edge and bulk at *B*=0T.

Spectroscopy of the edge and bulk as function of back-gate voltage at *B*=0T. Trivial behavior is seen. **a)** The edge is gapped. **b)** The bulk shows trivial ABS crossing.
